# Integrating Microbial Fuel Cell and Hydroponic Technologies Using a Ceramic Membrane Separator to Develop an Energy–Water–Food Supply System

**DOI:** 10.3390/membranes13090803

**Published:** 2023-09-19

**Authors:** Chikashi Sato, Wilgince Apollon, Alejandro Isabel Luna-Maldonado, Noris Evelin Paucar, Monte Hibbert, John Dudgeon

**Affiliations:** 1 Department of Civil and Environmental Engineering, Idaho State University, 921 S. 8th Ave., Stop 8060, Pocatello, ID 83209, USA; evelinpaucardelac@isu.edu (N.E.P.); montehibbert@isu.edu (M.H.); 2 Department of Agriculture and Food Engineering, Faculty of Agriculture, Autonomous University of Nuevo Leon, Campus of Agricultural and Animal Sciences, General Escobedo 66050, Nuevo Leon, Mexico; wilgince.apollon@uanl.edu.mx (W.A.); alejandro.lunaml@uanl.edu.mx (A.I.L.-M.); 3 Department of Anthropology, Idaho State University, 921 South 8th Avenue, Stop 8094, Pocatello, ID 83209, USA; johndudgeon@isu.edu

**Keywords:** *Allium tuberosum*, ceramics, hydroponics, microbial fuel cell, nutrient recovery, wastewater treatment

## Abstract

In this study, a microbial fuel cell was integrated into a hydroponic system (MFC-Hyp) using a ceramic membrane as a separator. The MFC-Hyp is a passive system that allows the transport of nutrients from wastewater in the microbial fuel cell (MFC) to water in the hydroponic vessel (Hyp) through a ceramic membrane separator, with no external energy input. The performance of this system was examined using potato-process wastewater as a source of energy and nutrients (K, P, N) and garlic chives (*Allium tuberosum*) as a hydroponic plant. The results showed that based on dry weight, the leaves of *Allium tuberosum* grew 142% more in the MFC-Hyp than those of the plant in the Hyp without the MFC, in a 49-day run. The mass fluxes of K, P, and NO_3_^−^-N from the MFC to the Hyp through the ceramic membrane were 4.18 ± 0.70, 3.78 ± 1.90, and 2.04 ± 0.98 µg s^−1^m^−2^, respectively. It was apparent that the diffusion of nutrients from wastewater in the MFC enhanced the plant growth in the Hyp. The MFC-Hyp in the presence of *A. tuberosum* produced the maximum power density of 130.2 ± 45.4 mW m^−2^. The findings of this study suggest that the MFC-Hyp system has great potential to be a “carbon-neutral” technology that could be transformed into an important part of a diversified worldwide energy–water–food supply system.

## 1. Introduction

As climate change concerns increase and the world population grows rapidly, the demands for renewable energy, freshwater, and food will surely rise, accompanied by the need for the treatment and reuse of wastewater. This study aimed to develop an energy–water–food supply system capable of generating electricity, producing edible plants, and treating wastewater simultaneously, using wastewater as a renewable source of electrical energy and plant nutrients. In current practice, wastewater is being treated in wastewater treatment facilities that employ energy-consuming methods [1,2,3], which release CO_2_ into the atmosphere. In recent years, wastewater (domestic, industrial, and agricultural) has been recognized as a renewable source of energy, freshwater, and nutrients because it primarily consists of water, energy-latent carbon compounds, and plant-growth nutrients [4,5,6,7,8,9].

Over the last several decades, bio-electrochemical processes have drawn considerable attention as they can be put into operation with low or near-zero energy input. In microbial fuel cells (MFCs), chemical energy contained in organic constituents is directly converted into electrical energy using exoelectrogenic or electroactive bacteria (EAB) as a catalyst, with little energy loss in the conversion process. Therefore, MFCs can generate electricity using wastewater as an energy source, while concurrently treating the wastewater [10,11,12,13].

Hydroponics is a form of hydroculture in which edible, medicinal, or ornamental plants are grown in nutrient-rich water without soil. In hydroponics, carbon and nutrients are captured and stored in plants as biomass, while oxygen is generated through photosynthesis and released into the environment [14,15]. Hence, hydroponics is considered an environmentally friendly technology that can be applied to wastewater remediation and water quality improvement [14,16,17,18]. One major advantage of hydroponics is that edible plants can be grown in a soil-less environment, such as in urban areas where cultivable land is limited, or arid regions where conservation of water is vitally important. Depending on the design and operational settings, highly valued plants can be grown at any time of the year. In the present study, *Allium tuberosum*, generally known as garlic chives or Chinese chives, was used as a hydroponic cultivar. *A. tuberosum* is a tough, fibrous, and edible perennial plant. It is easy to grow in different conditions, and it can adapt to various environments [19]. *A. tuberosum* has been widely cultivated for its culinary value in East Asia (e.g., China, Korea, Japan), Southeast Asia, and Northeast India [20]. It has also been used as a condiment and even as a traditional Chinese medicine [21,22]. Research has shown that it is rich in antioxidants and has antimicrobial activity [23]. Moreover, its seed oil has nutritional value [24,25].

Hydroponics is similar to a constructed wetland (CW) to a certain extent. The important difference is that the water in hydroponic systems must not be contaminated to grow edible, medicinal, or ornamental plants. On the other hand, CWs have been used to treat or polish treated wastewater (e.g., secondary effluent) by removing organics, nutrients, and suspended solids before being discharged to a receiving water body. Hardy aquatic plants (e.g., common reed and/or broadleaf cattail) are generally planted in or near water in a CW. In a constructed wetland-microbial fuel cell (CW-MFC) or sediment microbial fuel cell (sed-MFC), the electrodes are generally placed within the wetlands; in most cases, an anode is buried in the benthic sediment zone and a cathode is placed in the overlying water or in the plant root zone, as plants release O_2_ into the root zone through their aerenchyma tissue [26,27]. EAB in the sediment or anodic zone degrades organic matter to release protons (H^+^) into the anolyte while electrons are transferred to the anode electrode. While H^+^ migrates to the cathode, electrons flow through the external circuit to the cathode where O_2_ accepts electrons and H^+^ to form H_2_O. The plant roots at the cathode produce radial oxygen loss (ROL) that releases O_2_, which also serves as an electron acceptor [15]. Because of a lack of physical separation between the anode and the cathode in the CW-MFC, the diffusion of O_2_ into the anodic zone causes a significant loss of electrons, resulting in low power outputs [28]. A detailed review of CW-MFC is available in the literature [29].

Yadav et al. [30] designed and examined the wastewater treatment system called the integrated drip hydroponics-microbial fuel cell (IHP-MFC) using lemongrass (*Cymbopogon citratus*) as a hydroponic plant. Wastewater was recirculated through the system using a pump. Their system produced relatively low electrical outputs (maximum power density of ~32 mW m^−2^ normalized to the cathode area). The low electric power outputs were most likely attributed to the high resistance (0.12–20 kΩ) of non-conductive cocopeat used to support the plants and the diffusion of O_2_ from the aerobic (cathode) zone to the anaerobic (anode) zone, as a separator was not placed between them. Khuman et al. [28] developed the up-flow hydroponic/constructed wetland-microbial fuel cell (hydroponic CW-MFC) and evaluated it with and without a ceramic separator. In their system, the ceramic separator was sandwiched between the anode and cathode electrodes, and synthetic wastewater was pumped into the bottom of the system (anodic zone) and released from the top of the system (cathodic zone) where Indian shot (*Canna indica*) was grown. In their work, Khuman et al. [28] reported that the volumetric power densities produced by their system were 258.78 and 91.02 mW m^−3^ with and without the ceramic separator, respectively, and the internal resistances were 240 and 626 Ω with and without the separator, respectively.

Yang et al. [21] evaluated a system called an ecological floating bed-microbial fuel cell (EFB-MFC) with various aquatic plants, including windmill grass, goldfish algae, water hyacinth, and water spinach. In their work, EFB-MFC with water spinach produced the best performance with the highest voltage of 684 mV, the maximum power density of 6.03 mW m^−2^, and the lowest internal resistance of 94.33 Ω. The major drawback of the previous systems (e.g., IHP-MFC, hydroponic CW-MFC, and EFB-MFC) is that hydroponic plants were directly exposed to wastewater or contaminated water, which raises a public health concern if these systems were to be applied to hydroculture practices that produce edible or medicinal plants.

MFCs are structurally classified into two-chamber (or dual-chamber) and single-chamber MFCs. Descriptions of MFCs are available elsewhere [31]. The present study used an air-cathode single-chamber MFC. Because a cathode chamber and catholyte are not present in the air-cathode single-chamber MFC, operation and maintenance are easier compared to the two-chamber design [32]. Moreover, the reduction reaction of O_2_ at the cathode is ideal, as free oxygen (O_2_) is abundant in the air. However, the major disadvantage of the air-cathode MFC is that solids (precipitates) form on the cathode surface due to increased pH near the cathode and the permeation of anolyte into the cathode membrane followed by the evaporation of water. Past studies suggest that PO_4_^3−^ and NH_4_^+^ precipitate as struvite (MgNH_4_PO_4_·6H_2_O), other minerals such as Mg_3_(PO_4_)_2_·22H_2_O, MgKPO_4_, and potentially Ca_3_(PO_4_)_2_ [33,34,35]. The accumulation of these solids on the cathode surface requires frequent cleaning. Furthermore, to recover these solids as nutrients (fertilizer), the precipitates need to be scraped off the cathode surface. Such work is laborious and can damage the electrode.

In this study, the single-chamber MFC was placed in the hydroponic system to test the concepts that (1) nutrients (e.g., NH_4_^+^, PO_4_^3−^) will stay in ionic forms, as the cathode is always wet and the pH of the wastewater will not increase sufficiently to form and accumulate precipitates around the cathode, and (2) nutrients will diffuse from the MFC into the hydroponic system where the nutrients will be taken up (recovered) by the plant. At the same time, it is expected that organics in the wastewater decompose to release CO_2_, H^+^, electrons, and other end products in the MFC. Released CO_2_ dissolves into the wastewater to form carbonate species (e.g., HCO_3_^−^), which diffuse into the water in the hydroponic system where plants utilize them along with other nutrients. In return, the plants provide O_2_ to the MFC cathode as an electron acceptor. End-products of the MFC-Hyp are plant biomass, electricity, stabilized sludge, and treated wastewater with low organic and nutrient contents.

**Figure A1:**
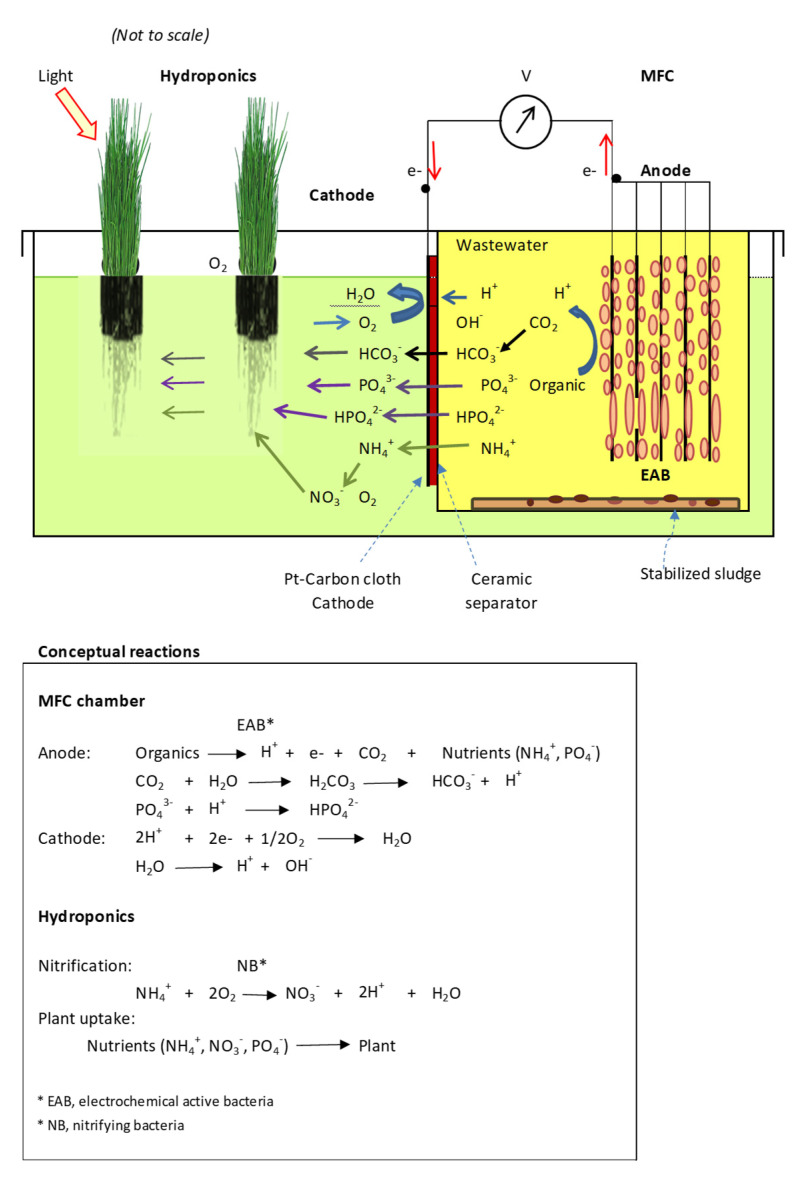
Conceptual MFC-Hyp system.

Hydroponics as a hydroculture system has been extensively studied; however, little work on the MFC-Hyp system with a ceramic membrane separator has been reported. This study aims to verify the concept of using the MFC-Hyp system to develop an energy–water–food supply system. The performance of the MFC-Hyp is examined based on the growth of the edible plant (*Allium tuberosum*) and the nutrient transfer rates and mass fluxes from the MFC to the hydroponic system through the ceramic membrane, in addition to measurements of MFC performance including the power output (maximum power density) and removal of organics (COD). This paper presents a proof-of-concept of the MFC-Hyp system operated in a laboratory environment as an energy–water–food supply system. A schematic of the conceptual MFC-Hyp system with potential reactions is presented in Figure A1 in Appendix A.

## 2. Materials and Methods

### 2.1. MFC Design

**Figure 1:**
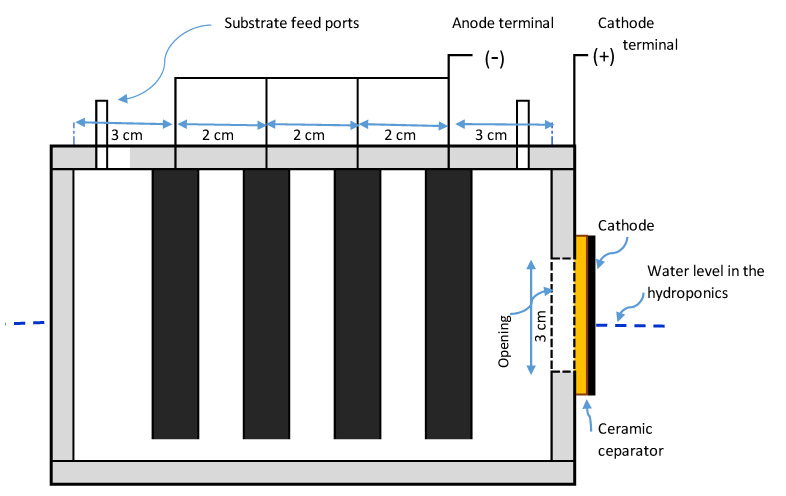
A single-chamber microbial fuel cell with a ceramic membrane separator.

Three units of the MFC-Hyp system were constructed to run concurrently. The three air-cathode single-chamber MFCs used in this study were described and characterized in our previous work [36]. In brief, the outside dimensions of each MFC are 13 cm long by 9 cm wide by 11 cm in height (Figure 1). The wastewater feed and removal ports (located on the top of the MFC) are obturated during the operation to maintain anaerobic conditions and also to prevent the direct release of CO_2_ into the atmosphere. A circular opening (3 cm diameter) was drilled into the side wall of the MFC chamber, which was covered by a cathode electrode and a ceramic membrane (thickness of 0.4 cm). The area of the opening is 6.7 cm^2^, which is considered the surface area of the cathode as well as the surface area of the ceramic membrane separator. The cathode electrode was made of carbon cloth with platinum loadings of 0.3 mg/cm^2^ (Fuel Cell Earth, Woburn, MA, USA). The anode electrode consisted of four pieces of low-resistance bamboo charcoal (Mt Meru Pte, Singapore). The characteristics of the bamboo charcoal anode are summarized in Table A1 in Appendix A.

### 2.2. Ceramic Membrane Separator

Two types of ceramic membranes were prepared according to Paucar et al. [18]. The Peruvian clay ceramic membrane was made of clay obtained from the Department of Junín, Peru. The craft shop clay ceramic membrane was prepared using clay obtained from the Craft Shop of Idaho State University in Idaho, USA. These ceramic membranes were imaged on an FEI Quanta 200 FEG scanning electron microscope. To obtain an image of the material porosity, each ceramic disk was broken along one edge to obtain a flat surface and mounted to a microscope stub using silver paste. Disk fragments were coated with gold-palladium in an SPI sputter coater to minimize sample charging during imaging. Each membrane was imaged at low (~1.3 mm HFW [horizontal field width]) and high (~200–400 µm HFW) magnification. Secondary and backscattered image inputs were combined in a composite image to better resolve both the surface texture and porosity. After careful observation of SEM images (which are presented in the discussion section), it was decided that Peruvian clay ceramic membrane be used as a separator in this study.

### 2.3. MFC-Hyp System

**Figure 2:**
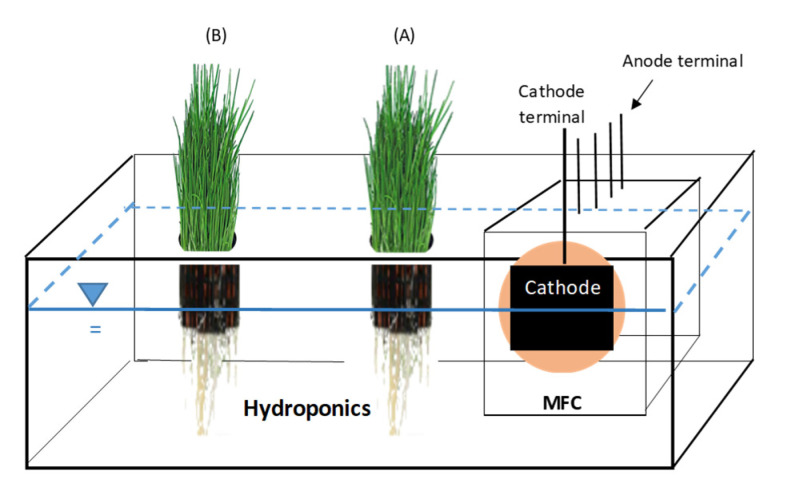
Microbial fuel cell integrated into hydroponics (MFC-Hyp): A lid is not shown.

Figure 2 shows the MFC-Hyp system in which the single-chamber MFC (shown in Figure 1) was integrated into the hydroponic system. The hydroponic vessel was made of a plastic container (33 cm long by 20 cm wide by 11.5 cm in height). An effective water volume of 2500 mL was maintained by periodically adding deionized (DI) water to the tank. On the lid of the tank, a rectangular opening (13.5 cm long by 9.5 cm wide) was made to house the MFC, and two circular openings (7.3 cm in diameter) were made for placing net pots to support hydroponic plants. The pots of the plants located closer to the MFC (6 cm from the cathode) and further (16 cm from the cathode) from the MFC were labeled A and B, respectively. The whole system, except plant leaves, was covered with cardboard to block light to prevent algae growth in the hydroponic water. Artificial light was provided in the area of the plants in a 12 h/day cycle using 5500 K Daylight Indoor Grow Lights (GHodec, Denver, CO, USA). Photo-synthetically active radiation (PAR) at a 400 to 700 nm wavelength was measured using a PAR meter (QPM-200, Quantum Sun, Annapolis, MD, USA). PAR (mean ± standard error; *n* = 48) was 8.89 ± 0.48 µmol m^−2^ s^−1^.

### 2.4. Bacterial Culture and Substrate Wastewater

Synthetic potato-process wastewater was prepared and used as fuel (this also served as an anolyte and substrate) for the MFC. To prepare the wastewater, a solution containing 0.5% potato extract and 0.1 M phosphate buffer was autoclaved for 20 min at 121 °C and stored at 4 °C until used. Concentrated potato extract was obtained from a local food processing plant in Idaho, USA. The bacterial culture was originally obtained from an anaerobic digester at the city of Pocatello wastewater treatment facility in Idaho, USA. The culture used in this study has been maintained in synthetic potato-process wastewater used in MFC reactors in the laboratory for many years and has been used in past MFC studies [36,37,38]. The bacterial community predominantly consists of *proteobacteria*, *firmicutes*, and *Bacteroidetes* [38]. It is noteworthy that, in the state of Idaho, USA, an estimated 10+ billion pounds of potatoes are harvested each year, of which approximately 62% are processed to make frozen and/or dehydrated potato products [39]. These processes require millions of gallons of water each year, which ends up in a waste stream. Idaho potato process industries must treat and dispose of their wastewater, which contains large amounts of organic matter and other constituents [40]. Because concentrations of the various constituents in the wastewater released from the industrial processes are irregular and unpredictable, synthetic potato-process wastewater was used in this study to achieve consistent and reproducible operation of the MFC-Hyp system.

### 2.5. MFC-Hyp System Operation and Monitoring

In the preparation stage, the MFCs were run outside the hydroponic system. After 2 weeks of the preparation run, the MFCs began to produce stable voltage. At this stage, the MFCs were emptied, filled with fresh wastewater, and placed inside the hydroponic vessels. Three sets of MFC-Hyp (namely MFC-Hyp 1, 2, and 3) with *A. tuberosum* were run concurrently for 49 days and repeated without *A. tuberosum*. The MFC-Hyp run without *A. tuberosum* represents the control run. All experimental runs were carried out in a batch mode at room temperature (22 ± 1 °C). Experimental conditions are summarized in Table A2 in Appendix A.

Plant growth parameters used in this study are the length and wet and dry masses of plant leaves. The plant biomasses were determined by the gravimetric method. In the preparation stage, *A. tuberosum* was grown in many net pots hydroponically in the laboratory for over a year. Prior to the experiment, nine pots containing plants with similar length and mass were selected. Two pots of the plants were placed in each hydroponic vessel, and the remaining three pots of the plants were used as the control in which no MFC was placed in the hydroponic vessel.

On Day 0 of the experimental run, a uniform cut was made to the leaves, leaving the stem with a height of 8 cm (as the base). Then, the two net pots containing the plants were placed in the hydroponic vessel, 6 cm and 16 cm away from the MFC cathode, which are indicated as pots A and B, respectively (see Figure 2). At the end of the run, plant height was measured from the base to the tallest point of the leaf, and the measurement was repeated three times. After the plant height measurements, the stem was cut to obtain the weight of the leaves (wet weight). The weights of the leaves in pots A and B were measured separately using an analytical balance. Subsequently, these leaves were dried in an oven at a moderate temperature of 65 °C until a constant weight was attained for each sample, generally 48 h to ensure that the residual water was evaporated completely. Finally, the dry weights of the leaves were measured using an analytical balance.

### 2.6. Wastewater and Water Sampling

Both hydroponic vessel water and MFC wastewater in the MFC-Hyp system were analyzed by monitoring water quality parameters, including pH, electric conductance (EC), chemical oxygen demand (COD), ammonium-nitrogen (NH_4_^+^-N), nitrate-nitrogen (NO_3_^−^-N), potassium (K), and phosphorus (P). Prior to water sampling, water in the hydroponic vessel was uniformly mixed using a stirrer (Nuova II, Thermolyne, Dubuque, IA, USA). Water samples were collected from the hydroponic vessels and analyzed at approximately 7-day intervals. All water samples were collected using a 50-mL syringe with a 0.2-μm filter (Millex-VV, Millipore, Molsheim, France). Wastewater in the MFCs was analyzed at the beginning and end of the run.

EC and pH were measured by a conductivity meter (HI8733 Hanna instruments, Smithfield, RI, USA) and a pH meter (Orion Star A121, Thermo Scientific, Waltham, MA, USA), respectively. The concentrations of inorganic elements (e.g., Na, K, P) were determined by ICP-MS (X-Series II, Thermo Scientific, Dreieich, Germany). The concentrations of COD, ammonium, and nitrate were determined by colorimetric/spectrophotometric methods using a spectrophotometer (Cary 3500 Multicell Peltier UV-Vis System, Agilent Technologies, Santa Clara, CA, USA). Methods from the literature were adapted and modified as necessary. COD was measured by the modified EPA standard method 5220 D using HACH digestion vials (HACH Company, Loveland, CO, USA). Ammonium (NH_4_^+^) was measured by the method described by Holmes et al. [41] and Solόrzano [42], which was modified as needed. Nitrate (NO_3_^−^) was measured by the EPA Method 352.1. All water samples were analyzed in triplicate unless otherwise stated. Microsoft Excel 16 was used for data processing. The statistical results are presented as the mean ± standard error unless otherwise noted.

### 2.7. Determination of Electric Power Outputs

The MFCs were connected to a data acquisition system (DAS) operated by LabVIEW software 2015 [37]. The anode and cathode electrodes of the MFC were connected through a 973-Ω external resistor, and the potential drop (V) across the external load (R_load_) was recorded continuously every 15 min. The resultant current (I) was calculated by Ohm’s law (I = V/R_load_), and the power (P) was calculated by P = I·V or P = V^2^/R_load_. The power density (PD) and current density (CD) were calculated by PD = P/Ac and CD = I/Ac, where Ac is the cathode surface area. Polarization was carried out by varying the external resistance from 20 to 2000 Ω. Internal resistance was determined based using the maximum power transfer theorem [43].

## 3. Results and Discussion

### 3.1. Wastewater in the MFC

Chemical characteristics of the synthetic potato-process wastewater are presented in Table 1, and wastewater quality (pH, EC, COD, NH_4_^+^-N, and NO_3_^−^-N) at the beginning and end of the run are presented in Table A3 in Appendix A. pH (mean ± standard error; *n* = 3) of the wastewater in the MFC alone (when the MFC was not integrated with hydroponics) was 6.88 ± 0.01 at Day 0. On Day 49, the pH of the wastewater was 8.04 ± 0.01 and deposition of solid precipitates was shown around the cathode. On the other hand, the pH of the wastewater in the MFC of the MFC-Hyp was 6.75 ± 0.13 on Day 49, and no deposition of precipitates appeared around the cathode. These results support concept 1: Nutrients (e.g., NH_4_^+^, PO_4_^3−^) stay in ionic forms, as the cathode is always wet and the pH of the wastewater does not increase sufficiently to allow the formation and accumulation of precipitates around the cathode. The results showed that the pH of the wastewater did not increase but rather slightly decreased over 49 days. The natural diffusion of water molecules between the MFC wastewater and the hydroponic water across the ceramic membrane was expected in a process similar to the osmosis that occurs through a semipermeable membrane. In the diffusion process, OH^−^ in the MFC wastewater could also diffuse to the hydroponic water, which is indicated by the slight increase in pH in the hydroponic water. Furthermore, over time, the concentration of phosphorus (P) increased from near zero to 1–4 × 10^4^ µg/L, and nitrate (NO_3_^−^-N) increased from nil to around 2 × 10^2^ µg/L in the hydroponic vessels. The concentrations of other ions (Na^+^ and K^+^) also increased in the hydroponic vessels. The only conceivable source of these ions is the wastewater in the MFC.

The values of wastewater EC in the MFC alone were 63.9 ± 0.1 and 92.5 ± 0.6 ms on Days 0 and 49, respectively. The increase in EC in the MFC was likely due to the decomposition of organics, which releases ionic species such as NH_4_^+^. The concentrations of ammonium (as N) in the wastewater in the MFC alone were 0.20 ± 0.02 and 0.40 ± 0.10 mg L^−1^ on Day 0 and Day 49, respectively. The increase in the ammonium concentration in the MFC was likely due to the ammonification of organic compounds in the wastewater.

EC values of the wastewater in the MFC of the MFC-Hyp without *A. tuberosum* were 63.9 ± 0.1 and 64.7 ± 2.2 ms on Day 0 and 49, respectively. No significant increase in EC of the wastewater indicates that certain amounts of ionic species diffused into the water in the hydroponic system.

Chemical oxygen demand (COD) was used as the indicator of the presence of organics in water and wastewater. The value (mean ± standard error; *n* = 3) of COD in the wastewater was 2673 ± 74 mg L^−1^ on Day 0. In the MFC of the MFC-Hyp, the COD values were 100 ± 9 and 604 ± 54 mg L^−1^ in the presence and absence of *A. tuberosum*, respectively, at Day 49. The results show that the removal of organics (as COD) in the MFC of the MFC-Hyp in the presence of *A. tuberosum* was 96.3%, which is considerably larger than 77.4% in the absence of the plant.

### 3.2. Water Quality in the Hydroponic Water

Temporal changes in pH and EC in the hydroponic water in the presence and absence of *A. tuberosum* are presented, respectively, in Figures S1 and S2, as supplemental information. Over the 49-day run period, the pH (mean ± standard error; *n* = 18) of the water in the hydroponic component of the MFC-Hyp was 7.38 ± 0.06 and 7.54 ± 0.08 in the presence and absence of *A. tuberosum*, respectively. In both cases, pH increased slightly with time; however, overall, pH was moderately stable in the hydroponic media. EC (mean ± standard error; *n* = 18) of the water in the hydroponic component of the MFC-Hyp was 1.55 ± 0.19 and 0.82 ± 0.16 ms in the presence and absence of *A. tuberosum*, respectively. Similar to pH, EC increased slightly with time. The differences in pH and EC between the two systems (in the presence and absence of *A. tuberosum*) may be associated with the excretion of plant root exudates and ROL. The differences in pH and EC between MFC-Hyp 1, 2, and 3 are likely due to the heterogeneity of clay and variations that occurred during the ceramic preparation process.

In the hydroponic media of the MFC-Hyp in the presence and absence of *A. tuberosum*, the values (mean ± standard error; *n* = 3) of COD were nil in all three units on Day 0, and 18.1 ± 0.8 and 17.9 ± 3.2 mg L^−1^, respectively, on Day 49. Hence, the COD level increased at a rate of 0.37 mgL^−1^d^−1^ in the hydroponic water with and without the plant. The presence of *A. tuberosum* did not significantly affect the COD level in the hydroponic water, although the plant exudes readily degradable small organic molecules such as sugars, amino acids, and other organic acids [44] and ROL releases O_2_ into the water [45]. It Is likely that the increase in COD in the hydroponic water was primarily due to the diffusion of oxidizable constituents from the MFC. Noting that the feed wastewater had a COD of >2600 mg L^−1^, the ceramic membrane effectively blocked the migration of organics from the MFC to the hydroponic water. The concentrations of ammonium (as N) and nitrate (as N) were, on average, 0.02 ± 0.01 and 0.41 ± 0.05 mg L^−1^, respectively, on Day 49. The higher concentration of nitrate relative to ammonium indicates that the ammonium ions (NH_4_^+^) that diffused through the ceramic membrane were quickly nitrified in the hydroponic water.

### 3.3. Power Output and Internal Resistance

**Figure 3:**
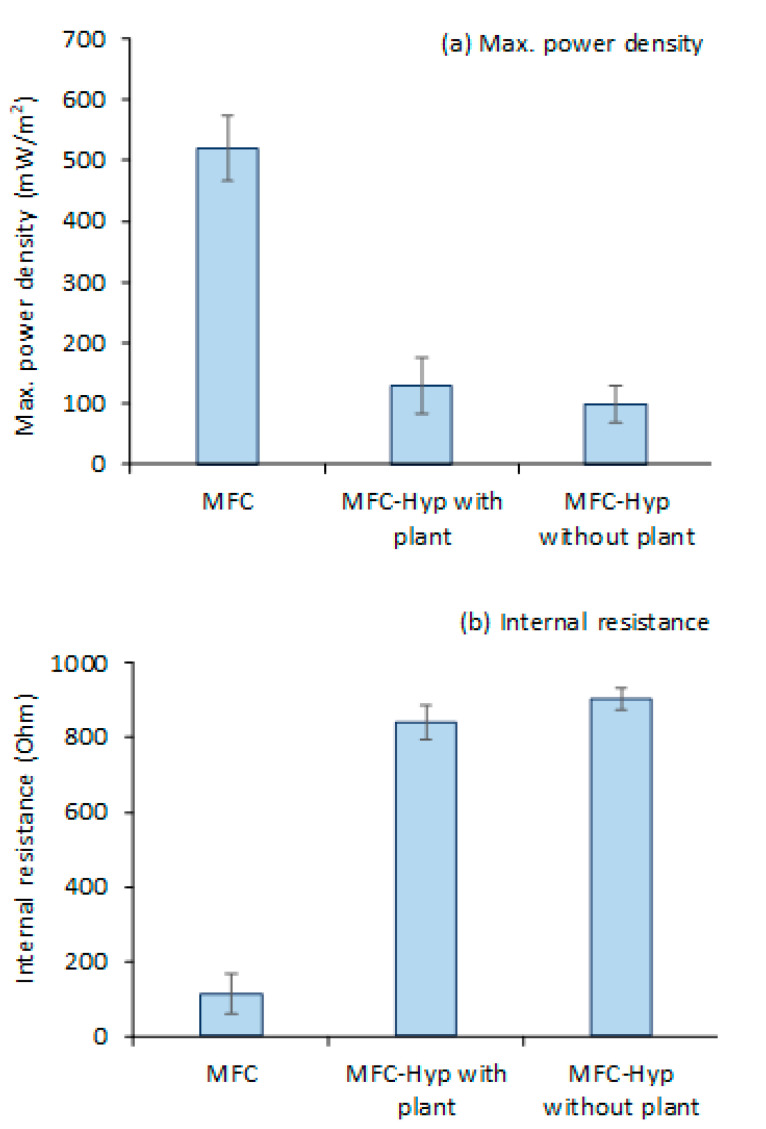
Power outputs and internal resistance of MFC, MFC-Hyp with and without plant (*Allium tuberosum*): (**a**) Maximum power density; (**b**) internal resistance.

Figure 3 shows the values (mean ± standard error; *n* = 3) of (a) the maximum power density and (b) internal resistance produced by the MFC alone, and the MFC-Hyp in the presence and absence of *A. tuberosum*. Polarization and power density curves for the MFC-Hyp 1, 2, and 3 in the presence and absence of *A. tuberosum* are presented in Figures S3 and S4, respectively, as supplemental information. The maximum power density produced by the MFC was 523.9 ± 50.0 mW/m^2^ at the maximum current density of 2676.7 ± 340.7 mA/m^2^. When the MFC was coupled with the hydroponic system, the maximum power density dropped significantly. The maximum power density produced by the MFC-Hyp was only 130.2 ± 45.4 mW/m^2^ with a current density of 558.0 ± 217.5 mA/m^2^ in the presence of *A. tuberosum* and 98.6 ± 30.2 mW/m^2^ with a current density of 417.8 ± 105.2 mA/m^2^ in the absence of the plant (Figure 3a). The significantly lower power outputs produced by the MFC-Hyp than that by the MFCs are associated with the larger internal resistance of the MFC-Hyp systems (Figure 3b). Because half of the cathode area was underwater in the MFC-Hyp, the availability of O_2_ at the cathode may have been limited. This resulted in an increase in the internal resistance of the MFC-Hyp system. A low dissolved oxygen (DO) level in the cathode region (aerobic zone) can result in the inefficient generation of electricity [46]. Conversely, high concentrations of DO in the cathode region can enhance power generation [22,47]. Without an effective barrier between the cathode and anode regions, DO in the cathode region could diffuse into the anode region to upset the anaerobic environment in the anode region. The presence of DO in the anode region inhibits the metabolic activities of EAB, resulting in reduced power output [48]. It is noteworthy that, in the presence of *A. tuberosum*, the maximum power density (130.2 ± 45.4 mW/m^2^) was 32% larger than that (98.6 ± 30.2 mW/m^2^) in the absence of the plant, and that the internal resistance (841± 264 Ω) of the MFC-Hyp in the presence of the plant was somewhat smaller than that (904 ± 172 Ω) in the absence of the plant. The enhancement of the MFC performance by introducing plants has been reported by other researchers [15,27,49]. The power outputs observed in this study and reported by other researchers are summarized in Table S1 as supplemental information. Yang et al. [27] studied the EFB-MFC with various aquatic plants. In their system, the anode was embedded into the sediment and the cathode was placed below the plant root zone. They reported that introducing plants into an MFC reduced the internal resistance of the system by 21.23–67.66% compared to the system without the plants. In the present study, *A. tuberosum* contributed to lowering the internal resistance of the MFC-Hyp. It is possible that ROL contributed O_2_ as an electron acceptor in the rhizosphere region [15,45].

Yadav et al. [30] operated the IHP-MFC system with 10 MFCs in series and parallel in a batch recirculation mode. Their system produced removal efficiencies of 72 ± 2.4% COD, 83 ± 1.1% phosphate, and 35 ± 4.2% ammonia after a 3 h run and a maximum power density of approximately 32 mW/m^2^ in series and parallel modes. The values of internal resistance were ~20 kΩ and 0.12 kΩ when MFCs were connected in series and parallel, respectively. The high COD removal rate and low power output suggest that their system experienced extensive diffusion of O_2_ to the anaerobic zone, as no separator was present between the anode and cathode electrodes. The high resistance of the cocopeat used to support the plants is also attributed to the low power density. In the study with the hydroponic CW-MFC system with *Canna indica* in the cathodic zone and 12.8 h wastewater residence time in the anodic zone, Khuman et al. [28] reported COD removal efficiencies of 86.2 ± 8.1% and 91.5 ± 4.9% with and without the ceramic separator, respectively. The volumetric power densities achieved by their system with and without the ceramic separator were 258.78 mW/m^3^ and 91.02 mW/m^3^, respectively, and the internal resistance values were 240 and 626 Ω, respectively. Similar to the IHP-MFC results, the smaller power output but larger COD removal efficiency indicate that there was an intrusion of O_2_ into the anode region. This occurs typically in the CW-MFC that has no separator. In the anode region, O_2_, if present, stimulates the growth of aerobic/facultative bacteria, which directly consume O_2_ (as an electron acceptor) to decompose organic matter, instead of transferring electrons to the anode electrode to generate electric current [50]. The larger power output but smaller COD removal efficiency is typically found in the system with the separator, as the separator blocks the diffusion of O_2_ into the anode region, creating higher anaerobicity (lower redox potential) and favoring the growth of EAB in the anodic zone [28].

### 3.4. Growth of A. tuberosum

**Figure 4:**
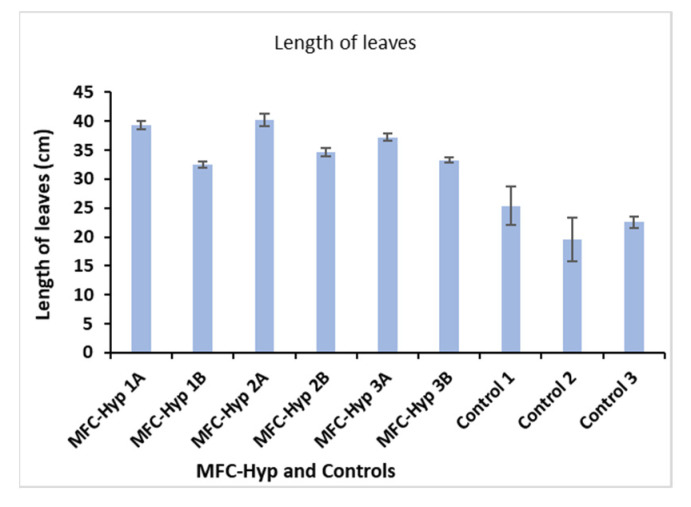
Change in the leaf length of *A. tuberosum*.

The growth of *A. tuberosum* was evaluated using three parameters: Length, and wet and dry weights of its leaves. The results are shown in Figure 4, where, for example, MFC-Hyp 1A and MFC-Hyp 1B indicate that the pots of the plants are located at the locations of A and B, respectively, in the MFC-Hyp 1 system. A comparison of the leaf length in the MFC-Hyp system and the hydroponic system alone (control) showed that the leaves in the MFC-Hyp systems are, on average 13.7 cm (61%) taller compared to those of the control. Furthermore, the leaves of the plants that are located at location A (closer to the ceramic membrane attached to the MFC) are 5.4 cm (16%) taller, on average, than those grown at location B.

**Figure 5:**
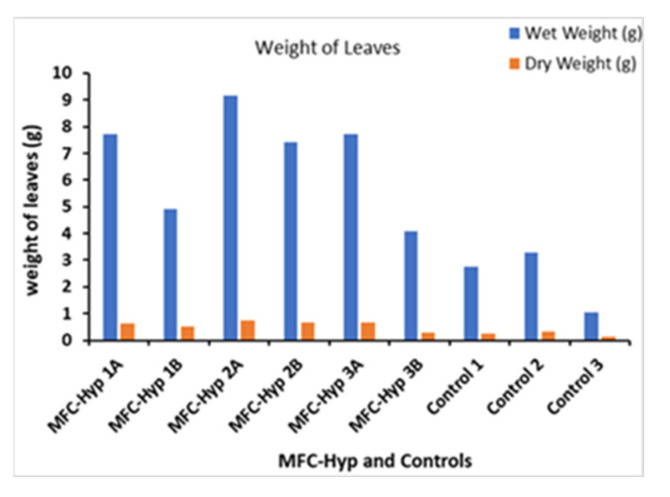
Change in the leaf weight of *A. tuberosum*.

Figure 5 shows the wet and dry weights of whole leaves of the plants grown for 49 days. The wet and dry weights of the leaves in the MFC-Hyp systems are, on average, 4.47 g (189%) and 0.34 g (142%) heavier than those of the control plants, respectively. Furthermore, the wet and dry weights of the leaves of the plants grown at site A are, on average, 2.74 g (50%) and 0.19 g (38%) heavier than those grown at site B, respectively. The results suggest that nutrients required for the growth of *A. tuberosum* diffused slowly from the MFC to the hydroponic system through the ceramic separator. In the IHP-MFC system developed by Yadav et al. [30], the growth rate of lemongrass (*Cymbopogon citratus*) was 216 ± 39 mg/month (7.2 ± 1.3 mg/day) based on the dry weight of leafy biomass per plant.

### 3.5. Nutrients in the Hydroponic Water

**Figure 6:**
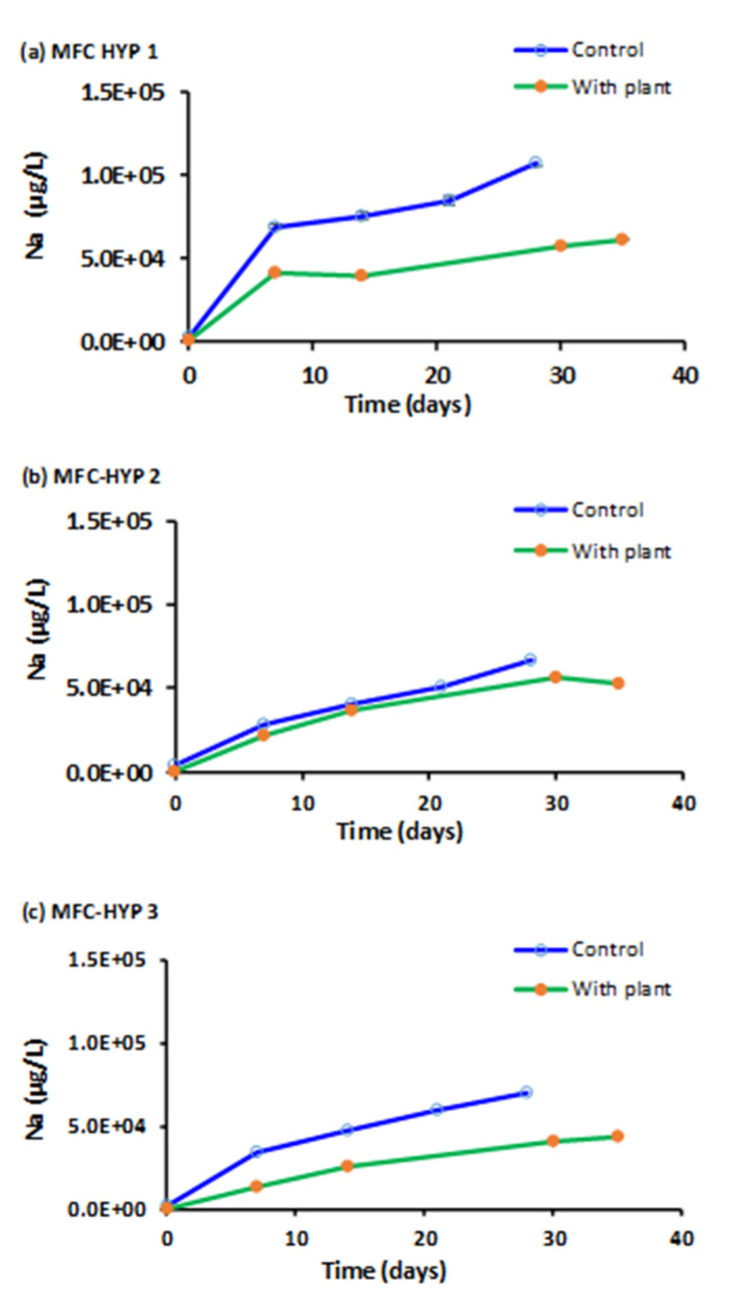
Sodium (Na) concentrations in the hydroponic water in the presence and absence of *A. tuberosum*: (**a**) MFC-Hyp 1; (**b**) MFC-Hyp 2; (**c**) MFC-Hyp 3.

**Figure 7:**
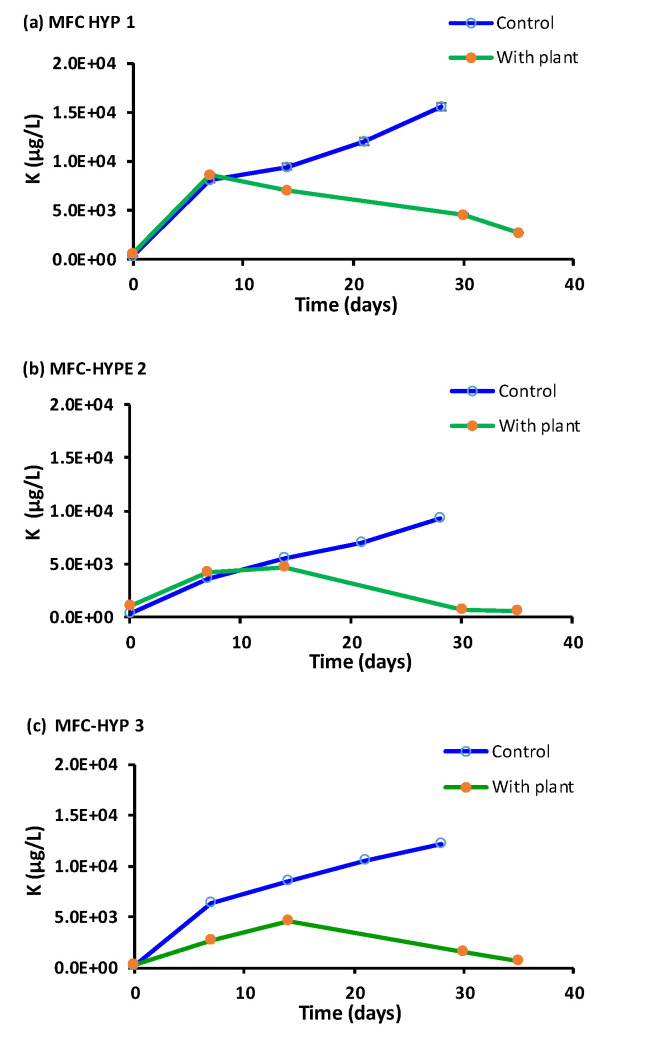
Potassium (K) concentrations in the hydroponic water in the presence and absence of *A. tuberosum*: (**a**) MFC-Hyp 1; (**b**) MFC-Hyp 2; (**c**) MFC-Hyp 3.

**Figure 8:**
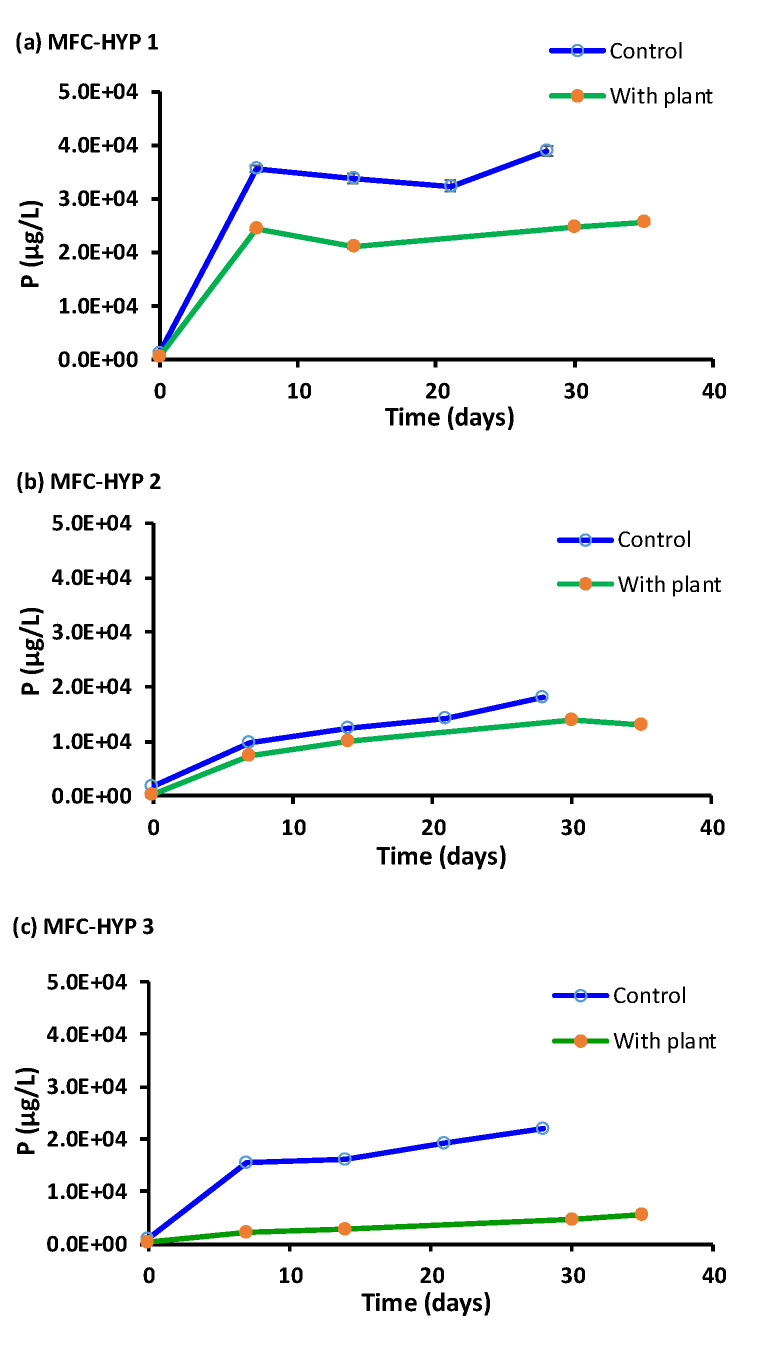
Phosphorus (P) concentrations in the hydroponic water in the presence and absence of *A. tuberosum*: (**a**) MFC-Hyp 1; (**b**) MFC-Hyp 2; (**c**) MFC-Hyp 3.

**Figure 9:**
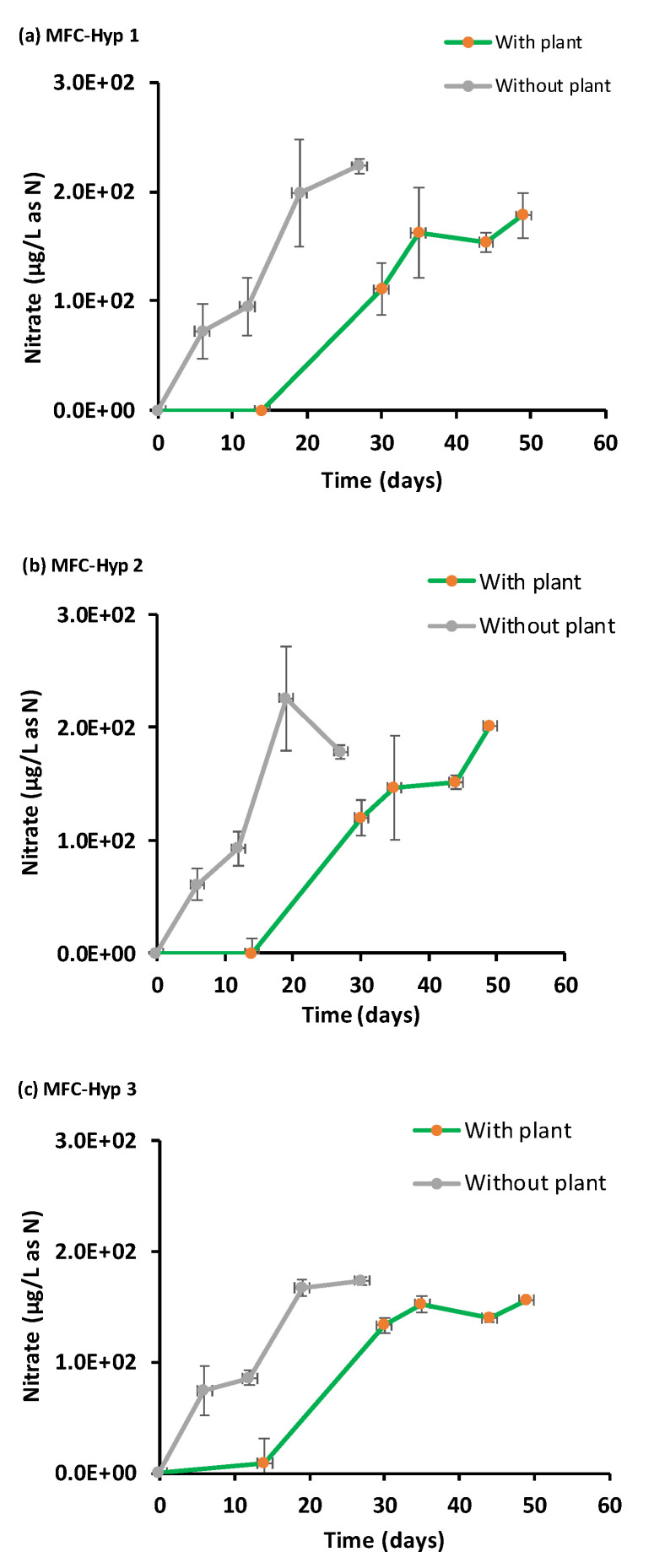
Nitrate (NO_3_-N) concentrations in the hydroponic water in the presence and absence of *A. tuberosum*: (**a**) MFC-Hyp 1; (**b**) MFC-Hyp 2; (**c**) MFC-Hyp 3.

In this study, particular attention was given to phosphorus (P), potassium (K), and nitrate as nitrogen (NO_3_^−^-N) as plant nutrients. Sodium (Na) was used as a reference, as Na^+^ is chemically stable and highly mobile in water. Figure 6, Figure 7, Figure 8 and Figure 9 show the concentrations of Na, K, P, and NO_3_^−^-N, respectively, in the hydroponic water of MFC-Hyp 1, 2, and 3 in the absence (control) and presence of *A. tuberosum*. The symbol represents the mean of duplicate measurements. The values of standard error are too small to be seen in Figure 6, Figure 7 and Figure 8. Note that, at the beginning of the run (Day 0), the concentrations of all ion species were nil (close to zero) as DI water was used in the hydroponic system. The Na concentration in the wastewater in the MFC was 3.06 × 10^6^ µg L^−1^ at Day 0. As seen in Figure 6, the Na concentration in the hydroponic water increased with time, indicating that Na migrated from the MFC to the hydroponic water. It was also observed that the Na concentration was lower in the presence of the plant than in the absence of the plant (although the concentration difference was small in MFC-Hyp 2 with and without the plant). The lower Na concentrations in the presence of *A. tuberosum* indicate that Na was taken up by the plant.

Figure 7 shows concentrations of K in the hydroponic water over time. The concentration of K in the wastewater in the MFC was 6.51 × 10^5^ ± 1.72 × 10^5^ µg L^−1^ at Day 0. In the presence of *A. tuberosum*, the concentration of K initially increased, but it began decreasing after approximately two weeks. Consequently, the difference of the K concentrations with and without the plant became larger with time. The results indicate that, as the plant grew, the rate of K uptake by the plant became larger than its diffusion rate through the ceramic membrane, and the growth of *A. tuberosum* was likely limited by K in the system.

As seen in Figure 8, the concentration profile of P is similar to that of Na, although the concentrations of P were considerably lower than those of Na. Note that the concentration of P in the wastewater in the MFC was 1.81 × 10^6^ ± 0.56 × 10^6^ µg L^−1^ at Day 0. While the concentration of P increased with time, its concentration was consistently lower in the presence of *A. tuberosum* than in the absence of the plant, indicating that P was taken up by the plant.

Figure 9 shows the temporal concentration of nitrate (NO_3_^−^-N) in the hydroponic water. The large error bars are due to the colorimetric method used in the nitrate analysis. The observed nitrate concentration increased over time both with and without *A. tuberosum*. The nitrate concentrations in both the absence and presence of *A. tuberosum* were nearly parallel, indicating that the MFC supplied enough nitrate to the hydroponic water to meet the N demand by *A. tuberosum*. The low concentration (10.0 ± 0.006 µg L^−1^) of ammonium (NH_4_^+^-N) in the hydroponic water suggests that ammonium was quickly nitrified to nitrate, followed by plant uptake. Also, ammonium can be taken up directly by the plant. These results support concept 2: Nutrients diffuse from the MFC to the hydroponic water where the nutrients will be taken up (recovered) by the plant.

As shown in Figure 7, Figure 8 and Figure 9, the concentrations of nutrients (K, P, N) in the hydroponic water were lower in the presence of the plant compared to those in the absence of the plant, suggesting that the nutrients were taken up by the plant. As the nutrients diffused from the MFC wastewater to the hydroponic water through the ceramic membrane, the concentration of the nutrient plume was expected to be more concentrated near the source (i.e., the ceramic membrane), and less concentrated away from the source. The results showed that the growth of the plant located near the ceramic membrane (location A) was greater than the plant located farther away from the ceramic membrane (location B). These results suggest that nutrients in the MFC wastewater slowly diffused through the ceramic membrane into the water in the hydroponic vessel. Consequently, the plant located at location A was exposed to higher concentrations of nutrients and produced more plant biomass. On the other hand, the plant located at location B was exposed to lower concentrations of nutrients, thus producing less biomass compared to the plant at location A.

### 3.6. Mass Transfer Rates

To quantify the diffusion of the nutrients from the MFC to the hydroponic water through the ceramic membrane, the mass transfer rates of Na, P, K, and NO_3_^−^-N were determined as follows. First, the mass of the selected constituent was calculated by

**Figure 10:**
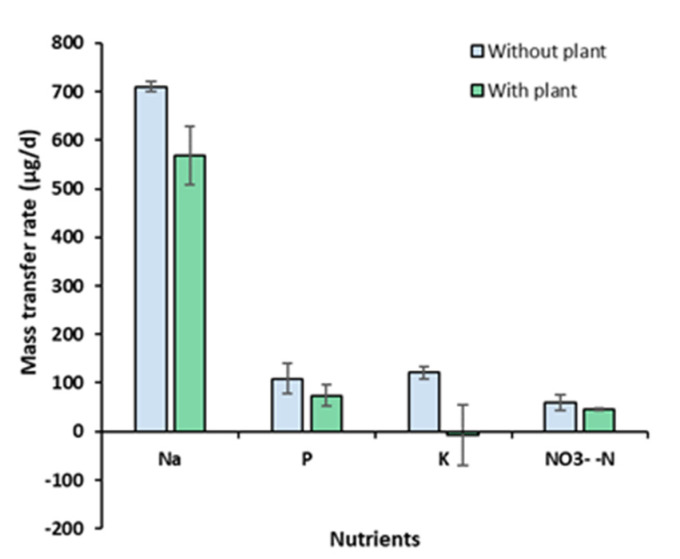
Mass transfer rate of Na, P, K, and NO_3_**^−^**-N from the MFC to the hydroponic water through the ceramic separator.

M_i,t_ = C_i,t_ V_Hyp_ (1)

where M_i,t_ = the mass of constituent i (µg) at time t; C_i,t_ = the concentration of the constituent i in the hydroponic water (µg L^−1^) at time t; and V_Hyp_ = the total volume of the hydroponic water (L). Second, the values of M_i,t_ were plotted as a function of time t (day), and a linear regression line was drawn. The slope of the line represents the mass transfer rate (µg d^−1^). The mass transfer rates (mean ± standard error; *n* = 3) determined for Na, P, K, and NO_3_^−^-N were 709.6 ± 10.6, 109.2 ± 31.7, 121.0 ± 11.8, and 13.3 ± 3.7 µg d^−1^, respectively, in the absence of *A. tuberosum* (control), and 568.6 ± 59.7, 73.9 ± 22.3, −6.7 ± 62.2, and 10.1 ± 0.5 µg d^−1^, respectively, in the presence of *A. tuberosum* (Figure 10). It is noteworthy that negative mass transfer rates were found for K in the presence of the plant. The difference in the mass transfer rates between MFC-Hyp with and without the plant indicates the plant uptake rate. Estimated plant uptake rates for Na, P, K, and NO_3_^−^-N are 141.0, 35.3, 127.6, and 3.2 µg d^−1^, respectively. The results show that the plant uptake rate for K (127.6 µg d^−1^) is larger than its transfer (diffusion) rate (121.0 µg d^−1^), resulting in a decreasing concentration of K in the hydroponic water, as is shown in Figure 7. This result indicates that the growth of *A. tuberosum* could have been limited by K.

As the mass transfer rate is expected to increase with increasing area of the separator, the mass transfer rate was normalized to the interface area of the ceramic membrane and presented as the mass flux (µg s^−1^m^−2^) in Table 2. In the control system without *A. tuberosum*, the mass fluxes of P, K, and NO_3_^−^-N are, on average, 3.77 ± 1.10, 4.18 ± 0.41, and 2.05 ± 0.56 µg s^−1^ m^−2^, respectively. These values are one order of magnitude smaller than that (24.52 ± 0.37 µg s^−1^ m^−2^) of Na. In the MFC-Hyp systems with *A. tuberosum*, the mass fluxes of P, K, and NO_3_^−^-N are 2.55 ± 0.77, −0.23 ± 2.15, and 1.55 ± 0.08 µg s^−1^ m^−2^, respectively, while the mass flux of Na was 19.64 ± 2.06 µg s^−1^ m^−2^. The variations of their values among MFC-Hyp 1, 2, and 3 are likely due to the heterogeneity of the clay and ceramic manufacturing conditions.

### 3.7. Ceramic Membrane Separator

The prevalence of numerous waterborne diseases throughout the world raises the need to protect our food from contaminated water. In order for the MFC-Hyp system to perform effectively, nutrient and carbonate species must diffuse from the MFC chamber to the hydroponic water, while other substances, especially pathogens, in the wastewater must be retained within the MFC chamber. In dual-chamber MFC studies, a variety of materials, mostly synthetic cation exchange membranes (e.g., nafion-117), have been employed to divide the anode and cathode chambers. The desired characteristics of these separators include high proton transport efficiency, biocompatibility, chemical stability, corrosion resistance, mechanical strength, environmental friendliness, and low cost [36]. Ceramic membranes have attracted considerable interest due to their excellent properties such as high fluxes, high durability, chemical, mechanical and thermal stability, bacterial resistance, ease of cleaning, availability, and long working lifetime [51].

**Figure 11:**
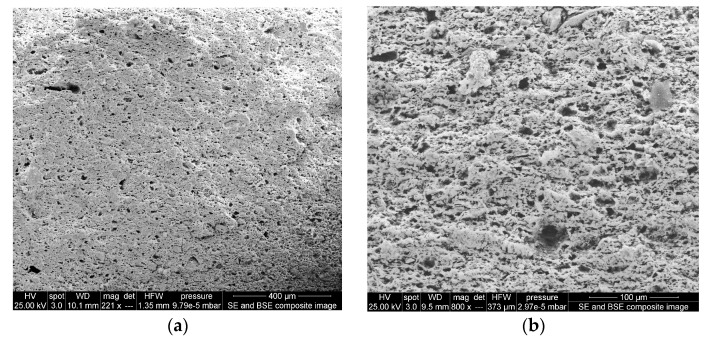

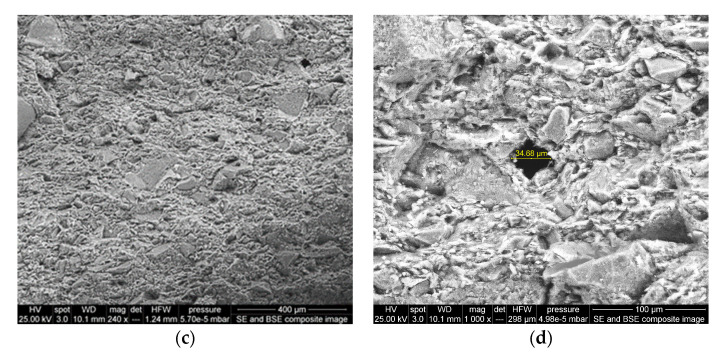
SEM images of ceramic membrane surfaces: (**a**,**b**) are Peruvian clay ceramic membrane, magnification of 221× and 800×, respectively; (**c**,**d**) are craft shop clay ceramic membrane, magnification of 240× and 1000×, respectively.

In the present study, two types of ceramic membranes were prepared: (i) A Peruvian clay ceramic membrane (Figure 11a,b), and (ii) a craft shop clay ceramic membrane (Figure 11c,d). These two ceramic clay membranes showed differences in their surface structures, with the craft shop clay being rather poorly sorted with a mixture of larger and smaller grains in the matrix and minimal observed porosity on the fractured surface. The Peruvian clay ceramic membrane had a well-sorted matrix with fewer large grains and a well-distributed surface porosity, with pore sizes predominantly in the 10–20 µm diameter range. Given the even distribution of the observed surface porosity, it is reasonable to presume the porosity of the Peruvian ceramic membrane is continuous throughout the matrix, providing intersecting channels, or circuits of sub-20 µm pores between both surfaces of the membrane disk, facilitating water and ionic solution passage, but blocking bacteria and other organisms from passing between the two chambers of the MFC-Hyp. Based on the observation of the SEM images, the Peruvian clay ceramic membrane was selected for use in this study.

The largest pore size of the Peruvian clay ceramic is approximately 20 µm, which is considerably larger than those investigated by Ghadge and Ghangrekar [52]. Their ceramic membranes were made of natural clay, natural clay containing 10%, and the same clay containing 20% Montmorillonite. They exhibited pore sizes of 2.14, 0.316, and 0.242 µm, respectively. Ghadge and Ghangrekar [52] reported that ceramic separators incorporated with Montmorillonite exhibited comparatively low resistance and less oxygen diffusion into the anodic chamber and ensured long-term stable MFC operation. As was stated previously, ceramics have many superior properties compared to polymer-based membranes. However, ceramics have a major drawback associated with the heterogeneity of clay (e.g., contents of Al_2_O_3_, ZrO_2_, TiO_2_, and SiC) and ceramic manufacturing conditions [53]. This leads to inconsistent performance with varying ion selectivity, permeability, and fouling. While attempts have been made to increase the power output of MFCs using a ceramic separator with different contents of montmorillonite and kaolinite [52], the comprehensive characterization and optimization of ceramic membranes are required to achieve consistent MFC-Hyp performance and estimate scale-up and implementation costs for full-scale applications.

In this study, a single-chamber MFC was integrated into a hydroponic system. In the MFC-Hyp, the MFC and the hydroponic system can complement each other by providing the following features:

Nutrient ions (e.g., K^+^, PO_4_^3−^, NH_4_^+^) present in wastewater diffuse from the MFC to water in the hydroponic system through a ceramic membrane due to the concentration gradient and/or electric field created by the MFC. Thus, the MFC-Hyp is a passive system that requires no external energy input.

- Because half of the cathode (along with the ceramic membrane) is submerged in the hydroponic water, an increase in pH of the anolyte near the cathode is buffered, which provides the following advantages: (i) Precipitation and deposition of minerals (e.g., struvite) on the cathode surface is prevented; (ii) precipitates, if formed, dissolve in water and are taken up (recovered) by the plant in the hydroponic system; (iii) cathode cleaning work is reduced; (iv) potential damage to the cathode due to cleaning is eliminated, providing a longer lifetime; and consequently, (v) the costs associated with operation and maintenance including nutrient recovery are reduced.
- Because half of the cathode area is exposed to water and the remaining half is exposed to the atmosphere, O_2_ in the water (from ROL) as well as in the air is available at the cathode as an electron acceptor. An optimum water-to-air area ratio of the cathode needs to be optimized in future research.
- It is expected that CO_2_ generated in the MFC dissolves in wastewater to form carbonate species (e.g., HCO_3_^−^). These species diffuse into hydroponic water and are taken by the plant. Consequently, CO_2_ emission into the atmosphere is reduced. This process will be confirmed and optimized in future research.
- In the integrated MFC-Hyp configuration, the MFC and the hydroponic system can be operated separately, which makes optimization of the system easier compared to a system where electrodes are placed within the hydroponic system.

The results presented in this paper serve as proof of principle and form a basis for expanding future research to optimize the process. There are challenges and unknowns to achieve full-scale design and implementation of the MFC-Hyp system. Nevertheless, the concept of a sustainable energy-water-food system by coupling MFC and hydroponic technologies was confirmed, although much research is still needed.

## 4. Conclusions

The results of this study support the concepts that (1) nutrients (e.g., NH_4_^+^, PO_4_^3−^) stay in ionic forms, as the cathode is always wet, and the pH of the wastewater does not increase sufficiently to cause the formation and accumulation of precipitates around the cathode, and (2) nutrients diffuse from the MFC to the hydroponic system where nutrients are taken up (recovered) by the plant. In the MFC, the concentration of organics (as COD) in the wastewater was significantly reduced and electricity was produced concurrently, although this process, especially the power output, requires improvement and optimization in future studies. The results of this study increase our understanding of MFC-Hyp technology. Once all the components are enhanced, refined, and optimized, the MFC-Hyp system can become an innovative new “carbon-neutral” technology that could be transformed into an important part of a diversified worldwide energy–water–food supply system.

## 5. Future Work

Although this study has demonstrated the concept of the MFC-Hyp system in the laboratory, further study is needed for real-world applications. Our priorities for future study include the following:

(1) Characterize and identify ceramic membranes that have the ability to selectively diffuse nutrients (P, N, and K), while effectively blocking harmful biotic as well as abiotic constituents so that clean and safe hydroponic water will be maintained. If needed, various minerals such as kaolinite and montmorillonite may be added to the base clay materials to improve ion selectivity and diffusability.
(2) Optimize the surface areas of the ceramic membrane and cathode electrode. It is expected that a larger surface area can provide increased nutrient transport to the hydroponic water; however, this must be balanced with the increased risk of intrusion of oxygen into the MFC wastewater, which reduces the MFC’s power generation efficiency.
(3) Improve the power output, including the innovation of the MFC design, the optimization of electrodes (materials, distance between the electrodes, sizes, etc.), and the selection of suitable wastewater.

## Figures and Tables

**Table 1:** Chemical characteristics of feed potato-process wastwater.

Parameters	Unit	Values *
pH		6.88 ± 0.01
Electric conductance	ms	63.9 ± 0.1
Chemical Oxygen Demand	mg/L	2673 ± 74
Ammonium-N	mg/L	0.20 ± 0.02
Nitrate-N	mg/L	0.38 ± 0.08
Phosphorus (P)	mg/L	1815 ± 558
Pottasium (K)	mg/L	651 ± 172
Sodium (Na)	mg/L	3060 ± 914
Magnesium (Mg)	mg/L	71.4 ± 15.7
Alminum (Al)	mg/L	14.28 ± 0.04
Sulfur (S)	mg/L	880 ± 112
Calcium (Ca)	mg/L	26.8 ± 2.3
Manganese (Mn)	mg/L	0.26 ± 0.07
Copper (Cu)	mg/L	0.30 ± 0.13
Zinc (Zn)	mg/L	73.93 ± 0.02

* Values are mean ± standard errior with *n* = 3 for pH, electric conductance, COD, ammonium-N, and nitrate-N, and *n* = 2 for all other inorganic elements.

**Table 2:** Mass flux (ug s^−1^m^−2^) of Na, P, and NO_3_-‒N in the MFC-Hyp with and without plant (*A. tuberosum*).

Nutrients	Without Plant	With Plant
	Mass Flux (µg s^−1^m^−2^) *	Mass Flux (µg s^−1^m^−2^) *
Na	24.52 ± 0.37	19.64 ± 2.06
P	3.77 ± 1.10	2.55 ± 0.77
K	4.18 ± 0.41	−0.23 ± 2.15
NO_3_^−^-N	2.05 ± 0.56	1.55 ± 0.08

* Mean ± standard error (*n* = 3).

## Data Availability

The corresponding author can provide the data upon request.

## Supplementary Materials

The following supporting information can be downloaded at: https://www.mdpi.com/article/10.3390/membranes13090803/s1, Table S1. Summary of different integrated systems for wastewater treatment, nutrient removal, and resource recovery (modified from [18]); Figure S1. pH and conductance of water in the hydroponics in the presence of *A. tubersosum*: (a) MFC-Hyp 1; (b) MFC-Hyp 2; (c) MFC-Hyp 3; Figure S2. pH and conductance of water in the hydroponics in the absence of *A. tubersosum*: (a) MFC-Hyp 1; (b) MFC-Hyp 2; (c) MFC-Hyp 3; Figure S3. Polarization and power density curves for MFC-Hyp 1, 2, and 3 in the presence of *A. tuberosum*; Figure S4. Polarization and power density curves for MFC-Hyp 1, 2, and 3 in the absence of *A. tuberosum*. Reference [54] is cited in the supplementary materials.

## Appendix A

**Table A1:** Dimensions and resistance of bamboo charcoal anodes (mean±standard error; *n* = 4).

MFC	Dimensions/BC Anode Plate				Resistance/BC Anode Plate		
	Length	Width	Thickness	Anode Area ^(a)^	Lengthwise	Widthwise	Average
	(cm)	(cm)	(cm)	(cm^2^)	(Ohm)	(Ohm)	(Ohm)
MFC 1	7.35 ± 0.13	4.28 ± 0.26	0.58 ± 0.10	73.76 ± 3.44	29.0 ± 2.74	23.3 ± 2.56	26.1 ± 2.63
MFC 2	7.25 ± 0.10	4.30 ± 0.11	0.78 ± 0.06	76.89 ± 1.65	25.0 ± 2.04	23.3 ± 2.10	24.1 ± 2.07
MFC 3	7.55 ± 0.13	4.00 ± 0.07	0.78 ± 0.05	75.17 ± 0.74	41.3 ± 7.51	38.8 ± 5.23	40.0 ± 5.38

BC, Bamboo charcoal anode; ^(a)^ Apparent anode area exposed to anolyte.

**Table A2:** Summary of experimental conditions.

Parameter	Value	Unit
Temperature *	22 ± 1	°C
MFC effective volume **	520 ± 10	mL
Hyp vessel effective volume **	2508 ± 13	mL
Hydroponic water depth **	6.25 ± 0.12	cm
Run duration	49	days
Flow scheme	Batch	

* Numerous observations; ** mean±standard error; *n* = 3.

**Table A3:** Wastewater quality at the beginning and end of the run.

Parameters	Unit	Beginning of the Run *	End of the Run *
pH		6.88 ± 0.01	8.04 ± 0.01
Electric conductance	ms	63.9 ± 0.1	92.5 ± 0.6
Chemical Oxygen Demand	mg/L	2673 ± 74	100 ± 9 **
Ammonium-N	mg/L	0.20 ± 0.02	0.40 ± 0.10
Nitrate-N	mg/L	0.38 ± 0.08	0.41 ± 0.05

* Values are mean ± standard errior with *n* = 3; ** With plant.
