# Supplementary material for: Integrating Microbial Fuel Cell and Hydroponic Technologies Using a Ceramic Membrane Separator to Develop an Energy–Water–Food Supply System
Source: Membranes (Basel). 2023 Sep 19;13(9):803. doi: 10.3390/membranes13090803 (PMC10538097; doi:10.3390/membranes13090803)
Supplement: Supplementary file 1 [file membranes-13-00803-s001.zip › membranes-2607761-supplementary.pdf]

Supplemental Information

Table S1. Summary of different integrated systems for wastewater treatment, nutrient removal, and resource recovery (modified from [18])

Type of System / Characteristics	Type of wastewater / Electrodes	Plant type / External resistance	Average Voltage mV	Max power density mW/m^2	Current density mA/m^2	CE %	COD Removal %	Nitrate Removal %	Phosphate Removal %	Plant biomass grow	Phosphate Recovery %	Reference
Two Upflow hydroponic CW-MFC (With ceramic separator, Without ceramic separator)<br><br>Continuous mode	Synthetic wastewater<br><br>Anode & cathode: carbon felts	*Canna indica*<br><br>1000 Ω	With ceramic separator: ~900 mV<br>Without ceramic separator: ~800 mV	With ceramic separator: 258.78 $mW.m^{-3}$<br><br>Without ceramic separator: 91.02 $mW.m^{-3}$	With ceramic separator: ~560 $mA.m^{-3}$<br><br>Without ceramic separator: ~190 $mA.m^{-3}$	NA	With ceramic separator: 86.2±8.1 %<br><br>Without ceramic separator: 91.5±4.9 %	NA	NA	NA	NA	[28]
Integrated drip hydroponics-MFC<br><br>Batch recirculation mode.	Domestic sewage collected from the sedimentation tank of the primary treatment unit<br><br>Anode& cathode: non-catalyzed disc-shaped graphite.	*Cymbopogon citratus*<br><br>20 kΩ	In series: 1490±91 mV<br><br>In parallel: 1580±5 mV	31.9 $mW.m^{-2}$ in series and parallel	In series: ~36 $mA.m^{-2}$<br><br>In parallel: ~458 $mA.m^{-2}$	NA	72±2.4% at HRT = 3 hours<br><br>85.7±0.6 % at HRT = 12 hours	NA	83.2±1.1 % at HRT = 3 hours<br><br>85.8±0.6 % at HRT = 12 hours	Per plant: 45±15 cm<br><br>0.216±0.039 g	NA	[30]

Table S1. (continued)

Type of System / Characteristics	Type of wastewater / Electrodes	Plant type / External resistance	Average Voltage<br>mV	Max power density<br>mW/m^2	Current density<br>mA/m^2	CE<br>%	COD Removal<br>%	Nitrate Removal<br>%	Phosphate Removal<br>%	Plant biomass grow	Phosphate Recovery<br>%	Reference
Floating treatment wetlands-MFC<br><br>Closed system for 3 weeks	Urban wastewater<br><br>Cathode: graphite rods<br><br>Anode: PVC hose filled with graphite sticks.	*Canna generalis*<br><br>*Chrysopogon zizanioides*<br><br>*Cyperus papyrus Nanus*<br><br>*Hymenachne grumosa*<br><br>*Equisetum hyemale*<br><br>1000 Ω	Maximum Voltages in:<br><br>Open circuit:<br><br>225 mV (*C generalis*)<br><br>212 mV (*H. grumosa*)<br><br>144 mV (*C. zizanioides*)<br><br>137.4 (*C. papyrus Nanus*)<br><br>89.6 mV (*E. hyemale*)<br><br>Closed circuit:<br><br>21.0 mV (*C generalis*)	0.93mWm^{-2} (When max voltage is 108 mV from all plants in parallel)	NA	NA	71.40%	TN: 8.4%	TP: 11.4%	NA	NA	[54]

Table S1. (continued)

Type of System / Characteristics	Type of wastewater / Electrodes	Plant type / External resistance	Average Voltage<br>mV	Max power density<br>mW/m^2	Current density<br>mA/m^2	CE<br>%	COD Removal<br>%	Nitrate Removal<br>%	Phosphate Removal<br>%	Plant biomass grow	Phosphate Recovery<br>%	Reference
Ecological floating bed-MFC	Synthetic eutrophication influent	*Cyperus alternifolius Linn.subsp. flabelliformis (Rottb.) Kukenth* (EFB-MFC1)	Control: 99mV				Control: 73.88%	TN:				
After 30 days start-up period, operated continuously for 116 days.	Anode & Cathode: stainless-steel mesh and carbon felt.	*Ceratophyllum demersum Linn* (EFB-MFC2)	EFB-MFC1: 125 mV				EFB-MFC1: 73%	Control: 38.74%<br>EFB-MFC1: 34.76%				
		Eichhornia crassipes (Mart.) Solms Pontereia crassipes Mart (EFB-MFC3)	EFB-MFC2: 144 mV	The maximum power density was EFB-MFC4: $6.03 mWm^{-2}$	NA	NA	EFB-MFC2: 76.37%	EFB-MFC2: 41.65%	NA	NA	NA	[27]
		Ipomoea aquatic Forsk (EFB-MFC4)	EFB-MFC3: 157 mV				EFB-MFC3: 78.23%	EFB-MFC3: 51.21%				
		500 Ω	EFB-MFC4: 161 mV				EFB-MFC4: 82.49%	EFB-MFC4: 55.6%				

Table S1. (continued)

Type of System / Characteristics	Type of wastewater / Electrodes	Plant type / External resistance	Average Voltage mV	Max power density mW/m^2	Current density mA/m^2	CE %	COD Removal %	Nitrate Removal %	Phosphate Removal %	Plant biomass grow	Phosphate Recovery %	Reference
MFC-Hyp-Plants<br>Batch mode.	Synthetic potato wastewater<br>Anode: bamboo charcoal plates.<br>Cathode: platinum-coated carbon cloth	*Allium tuberosum*<br>973Ω	Max 403mV	250.7	Max 2367.9	4.8	80.4±1.39	31.7±0.17	11.4±0.02	P1: 6.5cm<br>P2: 7.5cm<br>PC: 2.2 cm	7.3	[18]
MFC-Hyp-Control<br>Batch mode.	Synthetic potato-process wastewater<br>Anode: bamboo charcoal plates.<br>Cathode: platinum-coated carbon cloth.	*Allium tuberosum*<br>973Ω	Max 396mV	210.7	Max 1821.6	5.5	78.7±1.66	28.9±0.10	8.9±0.08	NA	4.7	[18]
MFC-Hyp<br>Batch mode.	Synthetic potato-process wastewater<br>Anode: bamboo charcoal plates.<br>Cathode: platinum-coated carbon cloth.	*Allium tuberosum*<br>973Ω	Max 344 mV	130.2±45.4 mW/m^2	558.0±217.5 mA/m^2	--	96.30%	-100% (Increased)	-7.9% (Increased)	142% (Dry weight)	--	This study

--, Not determined

NA, Not available

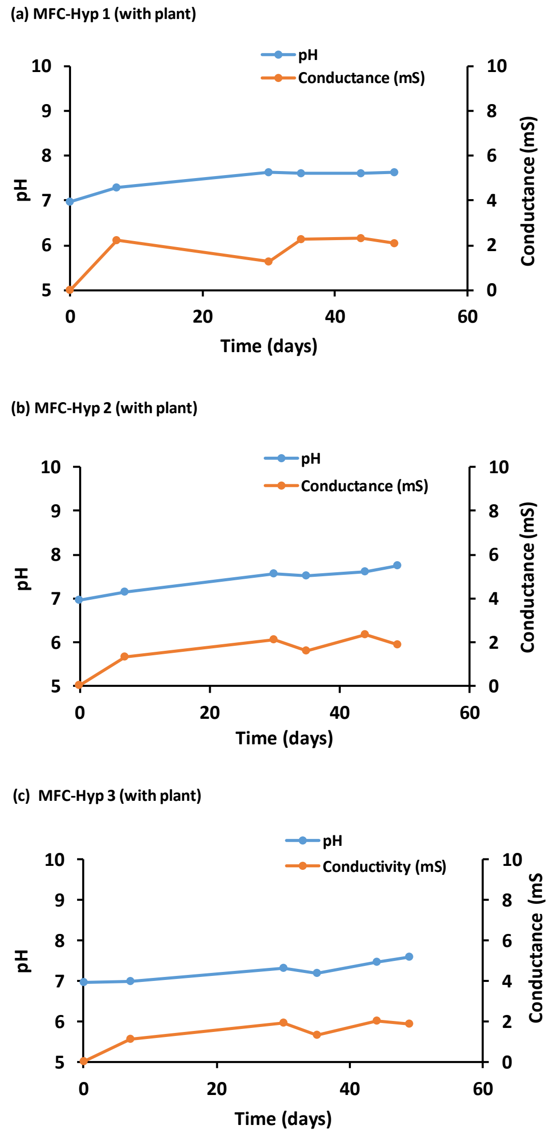

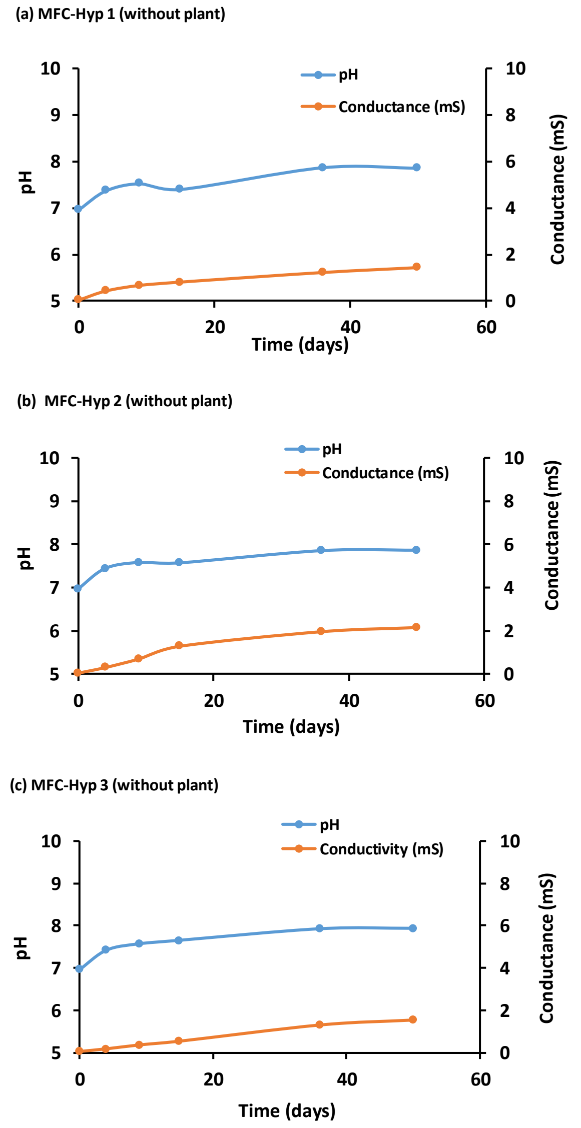

Figure S1: pH and conductance of water in the hydroponics in the presence of *A. tubersosum: (a) MFC-Hyp 1; (b) MFC-Hyp 2; (3) MFC-Hyp 3*

Figure S2: pH and conductance of water in the hydroponics in the absence of *A. tubersosum* : (a) MFC-Hyp 1; (b) MFC-Hyp 2; (c) MFC-Hyp 3

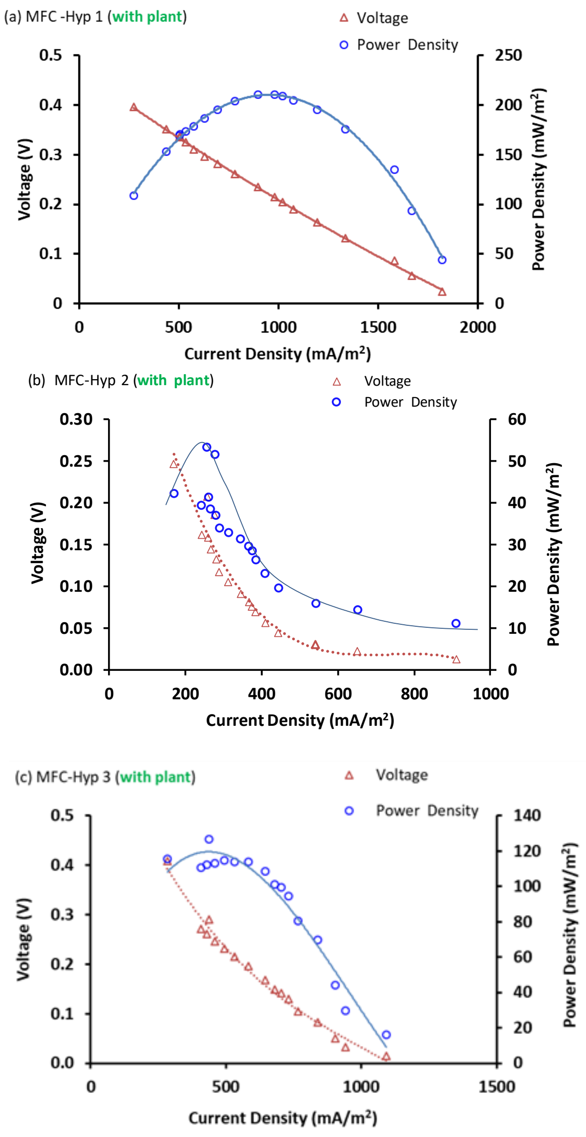

Figure S3. Polarization and power density curves for MFC-Hyp 1, 2, and 3 in the presence of *A. tuberosum.*

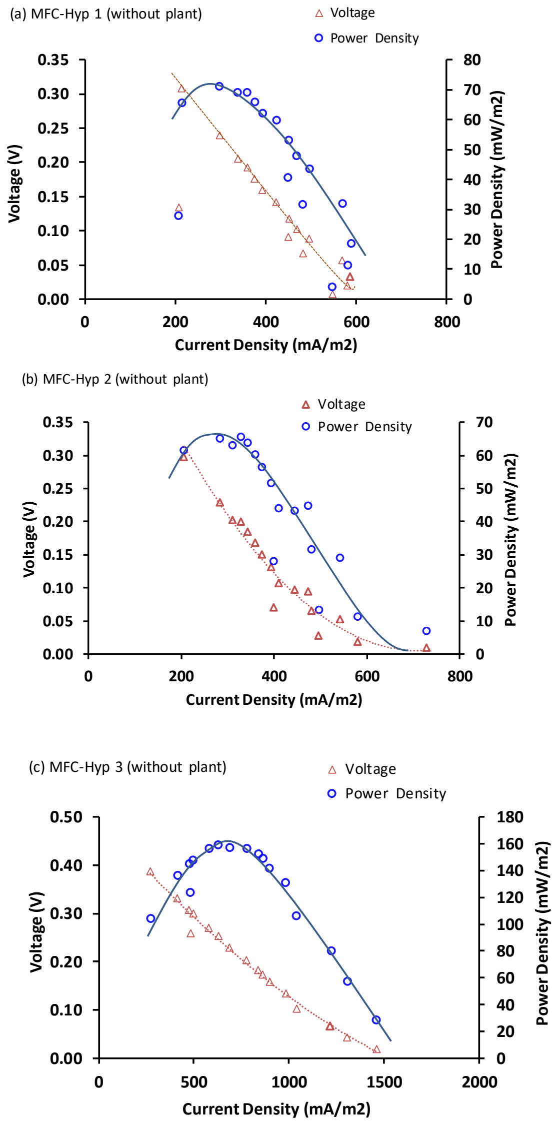

Figure S4. Polarization and power density curves for MFC-Hyp 1, 2, and 3 in the absence of *A. tuberosum.*
